# Evaluation of Endocrine Disruptome and VirtualToxLab for Predicting Per- and Polyfluoroalkyl Substances Binding to Nuclear Receptors

**DOI:** 10.3390/jox15050136

**Published:** 2025-08-22

**Authors:** Nina Franko, Manca Vetrih, Marija Sollner Dolenc

**Affiliations:** Department of Pharmaceutical Chemistry, Faculty of Pharmacy, University of Ljubljana, SI-1000 Ljubljana, Slovenia; nina.franko@ffa.uni-lj.si (N.F.); vetrih.manca22@gmail.com (M.V.)

**Keywords:** PFAS, Endocrine Disruptome, VirtualToxLab, endocrine disruption, androgen receptor, oestrogen receptor, glucocorticoid receptor, in silico, new approach methods

## Abstract

This study investigated whether the Endocrine Disruptome and VirtualToxLab in silico platforms are suitable for predicting the endocrine disrupting effects of per- and polyfluoroalkyl substances (PFASs)—in particular, for interactions with oestrogen receptors (ERs) and androgen receptor (AR). Compounds included in the U.S. Environmental Protection Agency’s PFAS working list were analysed with both models, and the results were compared with the available in vitro data regarding their modulation of nuclear receptors. Based on the identified prediction parameters, such as sensitivity, specificity, accuracy, and Mathews’ correlation coefficient, VirtualToxLab was found to be a reliable model for predicting the reactivity of PFASs with AR, while a positive consensus approach of both platforms provided reliable predictions of the PFAS reactivity with ERα and ERβ. This study provides the evidence that Endocrine Disruptome and VirtualToxLab can be used as a tier 1 screening tool for assessment of the endocrine disrupting effect of PFASs. Furthermore, it demonstrates that the likelihood of endocrine disrupting properties increases with the lipophilicity of PFASs and identifies the understudied PFHpS, PFNS, PFDS, 9-Cl, NMeFOSAA, NEtFOSAA, 4:2 FTS, 6:2 FTS, 8:2 FTS, 6:2 monoPAP, 8:2 monoPAP, and 5:3 acid as potential ligands of AR and/or ERs.

## 1. Introduction

Per- and polyfluoroalkyl substances (PFASs) are a family of synthetic chemicals that were introduced to the market in the 1930s due to their excellent resistance to high temperatures, pressure, and corrosive environments. To this day, PFASs are indispensable in most industries and are found in numerous products, including detergents, paints, cosmetics and personal care products, firefighting foams, and food contact materials (e.g., food packaging and baking paper) [1]. Their excellent resistance to harsh conditions is due to the extremely stable carbon-fluorine (C-F) bonds that also make them resistant to biodegradation [2]. Challenges in their elimination and their widespread use in various industries have led to them becoming a part of the exposome [3,4,5], resulting in a generalised exposure of humans, animals, plants, and the environment to PFASs.

Humans are ubiquitously exposed to PFASs [6] primarily through the consumption of drinking water and food, inhalation of contaminated air, exposure to contaminated soil and dust, and the use of PFAS-containing products [7,8]. The body’s ability to remove PFASs is limited, as they do not form metabolites and their long half-lives (e.g., up to 8.5 years for PFHxS) contribute to accumulation, particularly in the liver, lungs, serum, and kidneys [9]. The European Food Safety Authority (EFSA) recognises that adverse health effects, such as increased serum cholesterol levels and lower birth weight, are associated with exposure to PFASs [10,11]. Several studies have shown that PFASs also affect thyroid hormone levels [12,13,14], reproductive health in men and women [15,16], hypertension [17,18], and obesity [17,19]. PFOS and PFOA have been associated with reduced antibody response to vaccination, specifically to tetanus and diphtheria in both children and adults, and reduced antibody titres after influenza vaccination in adults [11]. This was recognised as a critical effect, leading the EFSA to set the weekly allowable intake of PFOA, PFNA, PFHxS and PFOS together at 4.4 ng/kg body weight in 2020 [11].

Several environmental pollutants, including bisphenols [20], pesticides [21], and PFASs [22], have been shown to exert harmful effects by acting as endocrine disruptors. Endocrine disrupting chemicals (EDCs) can mimic or interfere with the action of endogenous hormones, either by interacting with nuclear receptors (e.g., glucocorticoid receptor (GR) and androgen receptor (AR)), non-nuclear steroid hormone receptors (e.g., membrane-bound oestrogen receptor (ER)), non-steroidal receptors (e.g., dopamine receptor), or enzymes involved in steroid biosynthesis and/or metabolism [23]. According to the guidelines of the Organisation for Economic Co-operation and Development (OECD), the endocrine disrupting properties of chemicals should be investigated in a tier-based approach. In tier 1, the endocrine potential is assessed by analysing physicochemical properties, absorption, distribution, metabolism, and excretion (ADME) and predicting the structure–activity relationship, including in silico approaches. At tier 2, in vitro tests (e.g., transactivation tests and steroidogenesis) are performed, followed by in vivo tests at tiers 3 to 5 [24]. In line with tier 1, the European Chemicals Agency (ECHA) and the EFSA have issued a guidance document for the identification of EDCs [25], which lists several software tools for the prediction of endocrine activity, including the Endocrine Disruptor Knowledge Base (EDKB) [26], the OECD (Quantitative) Structure–Activity Relationships ((Q)SAR) Toolbox [27], the COSMOS KoNstanz Information MinEr (KNIME) workflow [28], Endocrine Disruptome [29], and VirtualToxLab [30]. Most of these tools are freely available and focus on predicting the reactivity of compounds with nuclear receptors. Endocrine Disruptome (ED) and VirtualToxLab (VTL) use a docking approach to assess reactivity with receptors, while the COSMOS KNIME workflow uses physicochemical and structural features to assess binding probability [29,30,31].

Today, in silico tools provide valuable data and offer a complementary approach to in vitro and in vivo testing, which can be time-consuming, expensive, and even ethically questionable. In particular, they can support the identification of the mechanisms of action and help to inform the decision on the most appropriate testing strategy [25]. When used as a tier 1 approach in the identification of EDCs, in silico tools should reduce the number of compounds to be tested in vitro and in vivo. In this case, it is assumed that all compounds identified as negative by the in silico tools are actually inactive against the tested targets, while the detected positive compounds are later subjected to in vitro and in vivo analysis to confirm or exclude their endocrine disrupting properties. Therefore, to obtain reliable results, the suitability of the in silico tool for the assessment of the selected class of chemicals must be checked. To date, the freely available in silico tools ED [29] and VTL [30] have not yet been analysed for their reliability in predicting PFAS interactions with nuclear receptors. The aim of this study is to compare their predictions of PFAS interactions with AR, ERs, GR, and PPARs with the available in vitro data and to evaluate their reliability by determination of prediction parameters such as sensitivity, specificity, and accuracy. In addition, while some PFASs (e.g., PFOS, PFOA, and PFHxS) have already been extensively studied for their endocrine disrupting effects [32,33,34,35], the safety of other substances (e.g., PFHpS, NMeFOSAA, and 4:2 FTS) has not yet been examined. These understudied PFASs have been assayed in this study using ED and VTL, providing new insights into their safety.

## 2. Materials and Methods

### 2.1. Selection of Compounds

The EPA’s PFAS working list (https://www.epa.gov/chemical-research/working-list-pfas-chemicals-research-interest-and-ongoing-work-epa, accessed on 23 April 2025), which contains compounds whose research is already underway, served as a basis for selecting compounds [36]. This list currently includes a total of 40 structurally different chemicals that fall into different PFAS groups (namely perfluoroalkyl carboxylates, perfluoroalkane sulfonates, perfluoroalkane sulphonamides, fluorotelomer alcohols, perfluoroalkyl ether carboxylates, and fluorotelomer phosphate esters). Seven PFASs were removed from the analysis, since the use of the ED is limited only to compounds with a molecular weight of up to 600 g/mol. The list of 33 compounds included in the analysis is shown in Supplementary Material, Table S1.

### 2.2. Docking with ED

Selected compounds were analysed using the docking programme Endocrine Disruptome (ED) [29], which can predict the binding affinities to the following nuclear receptors: AR, ERα, ERβ, GR, liver X receptors (LXRs) α and β, progesterone receptor (PR), peroxisome proliferator-activated receptors (PPARs) α, β, and γ, retinoid X receptor (RXR) α, mineralocorticoid receptor (MR), and thyroid receptors (TRs) α and β. For AR, ERα, ERβ, and GR, it can distinguish between agonistic and antagonistic conformations (abbreviated as an.). The molecules of interest were prepared as SMILE strings and inserted into ED (Table S1). Docking results were displayed as a score of predicted binding energy (kcal × mol^−1^) and the output was colour coded based on sensitivity (derived from thresholds specific to each receptor), with red (sensitivity < 0.25) representing high binding probability, orange (0.25 < sensitivity < 0.5) representing moderate binding probability, yellow representing (0.5 < sensitivity < 0.75) mild binding probability, and green (sensitivity > 0.75) representing low binding probability (criteria in Table S2). The analysis was repeated three times, and the arithmetic mean of the replicates was used to evaluate the tool. This in silico tool is suitable for the characterisation of molecules with a molecular weight of up to 600 g/mol, without multiple ionisation sites and without boron atoms. ED is freely available at http://endocrinedisruptome.ki.si/ (accessed on 23 April 2025).

### 2.3. Docking with VTL

VirtualToxLab provides an automated protocol for the simulation and quantification of small molecule binding to AR, ERα, ERβ, GR, LXR, PPARγ, PR, MR, TRα, TRβ, AhR, potassium ion channel (hERG), CYP1A2, CYP3A4, CYP2D6, and CYP2C9 [30]. The binding affinity output is expressed as the IC_50_ value, with a lower value representing a higher binding potential. If no interaction is predicted, VTL returns a result >100 µM. VTL is available free of charge to universities, government agencies, regulatory agencies, and non-profit organisations at https://pharma.unibas.ch/de/research/research-groups/computational-pharmacy-2155/research/virtualtoxlab/ (accessed on 23 April 2025). Compounds of interest were submitted to VTL as 3D.sdf files from the PubChem database and analysed for their binding affinity to AR, ERα, ERβ, GR, and PPARγ.

### 2.4. Database Search

#### 2.4.1. CompTox Database

The CompTox database [37] provides chemical properties, environmental fate, transport, hazard, in vitro to in vivo extrapolation, exposure, and bioactivity for over a million chemicals. It also includes data from TOX21 and ToxCast screenings, which represent screening assay data publicly available for prioritisation and hazard characterisation of thousands of chemicals, including PFASs. For the purpose of this study, the database was searched for the results of the TOX21 data obtained from the cell-based assays for the interactions of the substances with AR, ERα, GR, RXRα, PPARγ, and TR. The CAS numbers (Table S1) of the compounds of interest were entered into the CompTox database. The interactions with the receptors were searched and categorised as active or inactive based on the hit call. Interactions with nuclear receptors were considered positive if an active hit call was observed in any of the following assays:

- For AR:
  o TOX21_AR_BLA_Agonist_ratio
  o TOX21_AR_LUC_MDAKB2_Agonist
  o TOX21_AR_LUC_MDAKB2_Agonist_3µM_Nilutamide
  o TOX21_AR_BLA_Antagonist_ratio
  o TOX21_AR_LUC_MDAKB2_Antagonist_10nM_R1881
  o TOX21_AR_LUC_MDAKB2_Antagonist_0.5nM_R1881
- For ERα:
  o TOX21_ERa_BLA_Agonist_ratio
  o TOX21_ERa_LUC_VM7_Agonist
  o TOX21_ERa_LUC_VM7_Agonist_10nM_ICI182780
  o TOX21_ERa_BLA_Antagonist_ratio
  o TOX21_ERa_LUC_VM7_Antagonist_0.5nM_E2
  o TOX21_ERa_LUC_VM7_Antagonist_0.1nM_E2
- For ERβ:
  o TOX21_ERb_BLA_Agonist_ratio
  o TOX21_ERb_BLA_Antagonist_ratio
- For GR:
  o TOX21_GR_BLA_Agonist_ratio
  o TOX21_GR_BLA_Antagonist_ratio
- For PPARγ:
  o TOX21_PPARg_BLA_Agonist_ratio
  o TOX21_PPARg_BLA_antagonist_ratio

#### 2.4.2. PubMed Database

The results of ED and VTL were compared with the results of the literature search in the PubMed database. PubMed was searched for the combination of each chemical listed in Table S1 with the respective nuclear receptor (e.g., PFOSA and oestrogen; PFOSA and androgen; PFOSA and glucocorticoid; PFOSA and PPAR). Abstracts were screened to remove duplicates and review papers, and studies with binding assays or in vitro translocation assays capable of determining reactivity with AR, ERα, ERβ, GR, PPARα, and PPARγ were selected. In total, 26 articles were selected for the study (Table S4). Results were categorised as positive if at least two publications (including the CompTox database) showed positive reactivity of the compounds regardless of the effective concentration or if only one publication showed positive reactivity (e.g., significance, EC_50_, K_d_) at concentrations of 100 µM or less. Compounds that were found to enhance the activity of positive controls but were inactive when tested alone were categorised as negative.

### 2.5. Evaluation of ED and VTL

ED and VTL have been chosen for the evaluation since they are both recommended by the regulatory agencies for the assessment of endocrine disrupting chemicals [25]. Since they are freely available, easy to use, and able to assay large number of chemicals, they are frequently use in the field of in silico toxicology [38,39,40,41,42].

In agreement with previously published studies [43], the results predicted by ED were considered positive when the predicted binding energies in the agonistic and/or antagonist mode belonged to the red or orange class (high or moderate affinity), while the yellow and green classes were considered negative. With respect to VTL, results were considered positive if the predicted IC_50_ values were <100 μM. Results were categorised as true positive (TP) if the positive result of the in silico prediction matched the positive result of the in vitro tests. False positive (FP) results were determined when the in silico data showed positive results but the in vitro results were negative. True negative (TN) results were detected when the negative in silico predictions matched the negative in vitro results. If the negative results of the in silico predictions opposed the positive results of the in vitro data, they were categorised as false negatives (FNs).

Positive prediction values, negative prediction values, sensitivities, specificities, accuracies, and Mathew’s correlation coefficient (MCC) were calculated using Equations (1)–(6).

Positive prediction value (PPV) is the proportion of positive compounds correctly identified within all positive results and was calculated by Equation (1):

(1) $$PPV=\frac{TP}{TP+FP}\times 100\%$$

Negative prediction value (NPV) is the proportion of negative compounds correctly identified within all negative results and was calculated by Equation (2):

(2) $$NPV=\frac{TN}{TN+FN}\times 100\%$$

Sensitivity (Se) is the proportion of active compounds correctly identified and was calculated by Equation (3):

(3) $$Se=\frac{TP}{TP+FN}\times 100\%$$

Specificity (Sp) is the proportion of non-active compounds correctly identified and was calculated by Equation (4):

(4) $$Sp=\frac{TN}{TN+FP}\times 100\%$$

Accuracy (Acc) is the proportion of correctly identified compounds among all tested compounds and was calculated by Equation (5):

(5) $$Acc=\frac{TP+TN}{TP+FP+TN+FN}\times 100\%$$

Matthews’ correlation coefficient (MCC) describes whether the predictions are leaning towards perfect, random, or inverse and has been described as more reliable than Acc when analysing datasets with different numbers of positive and negative compounds [31,44]. MCC was calculated by Equation (6):

(6) $$MCC=\frac{\left(TP\times TN\right)-(FP\times FN)}{\sqrt{\left(TP+FP)\times (TP+FN)\times (TN+FP)\times (TN+FN\right)}}$$

An MCC score of +1 represents a perfect prediction, 0 an average random prediction, and −1 an inverse prediction.

### 2.6. Relationship Between Lipophilicity and Nuclear Receptor Interaction Potential

The lipophilicity of examined chemicals is described by the logarithm of partition coefficient (logP). For the purpose of this study, logP values were obtained from PubChem database [45] and are listed in Table S1. In order to examine the relationship between lipophilicity and endocrine disrupting properties, logP values were plotted against the number of nuclear receptors (AR, ERα, and/or ERβ) targeted by specific PFASs.

## 3. Results

### 3.1. Selection of Compounds and Receptors and Results of In Silico Analysis

The results of the in silico predictions with ED and VTL are listed in Table S3, and the results of the database searches are represented in Table S4. The databases yielded no data for PFPeS, PFHpS, PFNS, PFDS, PFOSF, NMeFOSSA, NEtFOSSA, 4:2 FTS, ADONA, PFMBA, PFEESA, PFMOPrA, 9-Cl, 8:2 FTS, 6:2 FTS, 6:2 monoPAP, 5:3 acid, or PFECA B. For the reactivity of the compounds with MR, PR, LXRs, TRα, and TRβ, there were limited or no in vitro data; therefore, these receptors were excluded from the analysis. Results of in silico analysis and corresponding in vitro assays that were used for the evaluation of ED and VTL are summarised in Table 1:

### 3.2. Evaluation of ED

Evaluation of ED with in vitro data (Table 2) revealed PPV values of 50% for AR, 100% for ERα, and 67% for ERβ. As shown by the PPV of 50%, ED tended to recognise AR-inactive PFASs as positive, resulting in a high proportion of FPs. On the other hand, the higher PPVs for ERs illustrated a low proportion of FPs for these receptors. The PPVs for GR, PPARα, and PPARγ could not be calculated, as ED did not find any positive hits for these targets. The ED performed well in identifying positive hits, as shown by the Se values for AR (100%), ERα (75%), and ERβ (6%). On the other hand, the Se values for PPARα (0%) and PPARγ (0%) indicate that it was likely to miss the actual positive hits and identify them as negative predictions.

Compared to the PPVs, the NPVs were higher for AR (100%) and ERβ (71%) but lower for ERα (75%). The NPV for GR was 100%, while the values for PPARα (21%) and PPARγ (44%) were much lower, as the ED recognised all true positives as FN. No FPs were detected for ERα, GR, PPARα, or PPARγ, indicating a 100% Sp, while a relatively high Sp was also detected for ERβ (71%). However, the Sp for AR was only 22% due to the high proportion of FP results mentioned above.

Acc and MCC are parameters that take into account all values determined (TP, FP, TN, FN). The highest Acc was found for GR (100%), followed by ERα (86%), ERβ (69%), and AR (56%), indicating that the majority of cases were correctly identified. On the other hand, the predictions for PPARα (31%) and PPARγ (44%) were worse, as Acc shows. In turn, MCC discriminates between inverse (values towards −1), random (values towards 0), and perfect predictions (values towards 1) better than Se, Sp, and Acc, especially when there is a different number of positive and negative predictions [31,44]. ERα showed the highest MCC of 0.8, indicating that the results tended towards perfect predictions. On the other hand, ERβ and AR showed MCCs of 0.4 and 0.3, respectively, suggesting that their predictions tended to be random. MCCs for GR, PPARα, and PPARγ could not be calculated, as no TPs or FPs were available.

### 3.3. Evaluation of VTL

The evaluation of the VTL (Table 3) resulted in a PPV of 100% for ERα, followed by 67% for AR, 62% for PPARγ, and 50% for ERβ. The PPV for GR was equal to 0, as no actual positive results for GR were found in the literature screen (Table S4). AR and PPARγ had high Se (over 85%) and thus a low proportion of FNs, meaning that the majority of positive hits were correctly identified. The Se were lower for ERα (25%) and ERβ (33%), suggesting that the actual positive compounds for these two receptors may be misjudged by VTL.

The NPVs were the highest for GR (100%), followed by AR (86%), ERβ (60%), ERα (50%), and PPARγ (67%). AR, ERα, and ERβ were accompanied by relatively high Sp (over 67%), indicating that the proportion of FPs was low. In contrast, GR and PPARγ showed low Sp (below 20%), which means that the actual negative results could be lost if PFASs are analysed with VTL for the reactivity with these two receptors.

The analysed data for the interactions of PFAS with AR by VTL also showed a high Acc of 75% and an MCC of 0.5, indicating a middle ground between random and perfect predictions while maintaining high accuracy. ERα, ERβ, and PPARγ had an Acc of 57%, 57%, and 63% and an MCC of 0.4, 0.1, and 0.2, respectively. In the case of ERα, it is hypothesised that the predictions were slightly more random compared to AR, but still tended towards the correct predictions. On the other hand, although ERβ and PPARγ had Acc values comparable to those of ERα, their MCC values were low, suggesting that the predictions were random. A low Acc of 18% was observed for GR, while its MCC could not be calculated due to the absence of TPs and FNs.

### 3.4. Combined Approaches

Previous studies have shown that the combination of two or more models results in improved prediction parameters [31,43,46]. Specifically, the “majority rule” and “positive consensus” approaches have been shown to improve Sp, Acc, and MCC, while the “negative consensus” approach can majorly improve Se [31,43]. Therefore, we investigated whether predictions could be improved by combining ED and VTL. First, we used a positive consensus approach, where predictions were considered positive if one or both models predicted positive outcomes. Second, the negative consensus approach was used, where predictions were considered negative if one or both models predicted a negative outcome. As the interactions of PFASs with PPARα are not predicted by VTL, this receptor was excluded from the analysis.

In a positive consensus model (Table 4), predictions for AR were equal to those of ED, while they were worse compared to VTL since more FPs were identified. As a result, Acc (56% vs. 75%) and MCC (0.3 vs. 0.5) were lower than in the VTL model. On the other hand, the positive consensus approach improved the predictions for ERα. All parameters were improved compared to ED and VTL itself, resulting in an Acc of 93% and an MCC of 0.9, indicating high reliability of the results. In the case of ERβ, there was a slight improvement compared to ED in terms of Se (83% vs. 67%), NPV (83% vs. 71%), Acc (71% vs. 69%), and MCC (0.5 vs. 0.4), while PPV (63% vs. 67%) was comparable and Sp (63% vs. 71%) was reduced. Compared to VTL, all parameters were significantly improved. The prediction parameters for GR worsened compared to ED due to the increase in FPs, leading to a decrease in Acc from 100% to 27%. Compared to VTL, the detection of TN was increased from 2 to 3, but the change was not sufficient to achieve reliable Acc (27%). The results of the PPARγ model were similar to those of the VTL model, with an improved PPV (62%), Se (89% vs. 0%), and Acc (60% vs. 40%) compared to ED, but with an MCC of 0.1, indicating low reliability of the results.

In the negative consensus model (Table 5), the parameters for AR were equal to those of VTL. Compared to the predictions by ED, PPV (67% vs. 50%), Sp (67% vs. 22%), Acc (75% vs. 56%), and MCC (0.5 vs. 0.3) were improved, while Se and NPV were reduced from 100% to 86%. Negative consensus led to poorer prediction parameters for ERα and ERβ compared to the two models, which even tended towards inverse predictions for ERβ (MCC −0.1). The results of GR and PPARγ were identical to those of the ED model.

The evaluation of ED, VTL, and a combination revealed that each model has its own strengths and limitations. The most reliable results for assessing the interactions of PFAS with AR were obtained with VTL, and the interactions with ERs were best predicted with the positive consensus approach of both in silico models. Therefore, we applied the term “ED/VTL approach” for the prediction of the interactions with the mentioned method. Since we were not able to investigate whether the actual GR binders can be correctly identified with ED and VTL due to the lack of positive in vitro data and since the predictions for PPARs were found to be unreliable, we do not recommend the use of the “ED/VTL approach” for these nuclear receptors.

### 3.5. Assessment of PFAS Reactivity with AR, ERα, and ERβ Using “ED/VTL Approach”

As shown in the previous sections, the “ED/VTL approach” is a valuable tool to investigate the interactions of PFASs with AR, ERα, and ERβ. Since there are no in vitro data available regarding the reactivity of PFMOPrA, PFPeS, PFHpS, PFNS, PFDS, 9-Cl, NMeFOSAA, NEtFOSAA, 4:2 FTS, 8:2 FTS, 6:2 FTS, ADONA, PFMBA, PFEESA, 6:2 monoPAP, 5:3 acid, or PFECA B with AR or ERs, these molecules were subjected to in silico analysis with ED and VTL (Table S3). In case of disagreement between the results obtained with the models, we followed the best prediction practises outlined in the sections above. Therefore, the decision for AR was made according to the predictions of VTL, while ERα and ERβ were evaluated according to the positive consensus procedure. The results are shown in Table 6 (in italics) and Table S3. In order to establish a structure–activity relationship between PFASs and their reactivity with nuclear receptors with greater confidence, the available results of the in vitro assays of PFBA, PFPeA, PFHxA, PFHpA, PFOA, PFNA, PFDA, PFUnA, PFBS, PFHeS, PFOS, PFOSA, 6:2 FTOH, 8:2 FTOH, HFPO-DA, and 8:2 monoPAP are also included in Table 6 (in bold).

As shown in Table 6 in bold font, most of the perfluoroalkyl carboxylates and fluoromer alcohols as well as one perfluoroalkane sulfonamide on the EPA’s PFAS working list have already been analysed in vitro, while the groups of perfluoroalkane sulfonates, N-alkyl perfluoroalkyl sulfonamido carboxylates, perfluoroalkyl ether carboxylates, fluorotelomer phosphate esters, fluorotelomer carboxylates, and perfluoroalkyl polyether carboxylates are yet to be analysed. Since the groups of perfluoroalkyl carboxylates (n = 9) and perfluoroalkane sulfonates (n = 8) are the largest, they are best suited for the evaluation of the structure–activity relationship.

**Figure 1:**
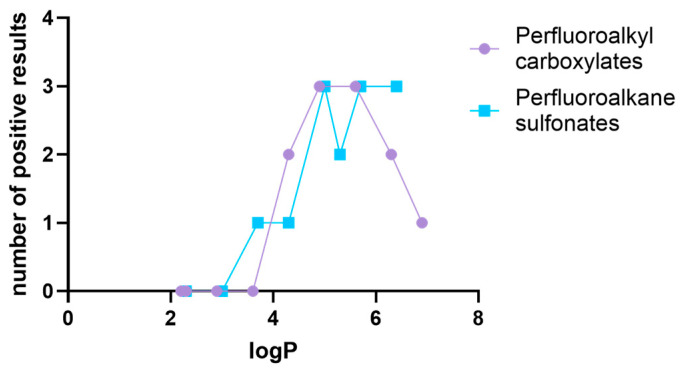
Relationship between PFAS lipophilicity and number of positive results for interactions with AR, ERα, and ERβ.

Comparison of the logP values (Table S1) of the compounds, with a number of positive results for AR, ERα, and ERβ interactions, shows that higher lipophilicity was associated with more interactions (Figure 1). This trend was more pronounced in the case of perfluoroalkane sulfonates. 9-Cl, with a logP of 5.3, seemed to follow this trend poorly, with only two predicted interactions. However, this was the only chlorinated compound among the sulfonates tested (see Table S1); therefore, different interactions with the receptors can be expected.

Within the perfluoroalkyl carboxylates, interactions with AR, ERα, and ERβ were observed for PFHpA (2 interactions), PFOA, and PFNA (3 interactions), with logP ranging from 4.6 to 5.6. Although PFDA and PFUnA exhibited a logP above 6, they reacted with a smaller number of nuclear receptors compared to less lipophilic analogues, indicating that longer chains of perfluoroalkyl carboxylates are unfavourable for interactions with AR, ERα, and ERβ. However, it must be noted that the positive/negative results regarding PFDA and PFUnA were obtained from the in vitro assays (Table S4) [35,37,47], but if judged using the “ED/VTL approach,” both PFDA and PFUnA are categorised as positive for interactions with AR, ERα, and ERβ (see Table S3). Based on this observation, it is surmised that VTL and especially ED tend to overestimate the reactivity of perfluoroalkyl carboxylates with AR and ERs. However, as only a limited number of in vitro studies have investigated the effects of PFDA and PFUnA [35,48], the decrease in endocrine disrupting properties due to increased lipophilicity should be treated with caution.

Judged by Table 6, 44.4% of perfluoroalkyl carboxylates were found to interact with AR and ERα and 33.3% with ERβ, while in the case of perfluoroalkane sulfonates, 62.2% of chemicals were found to be AR and ERβ binders and 50% of them reacted with ERα. These observations suggest that perfluoroalkane sulfonates exhibit generally higher potential for endocrine disruption, particularly for reactivity with ERβ.

PFOSA is the only perfluoroalkane sulfonamide on the working list, and judging by the Comptox database, it interacts with AR, ERα, and ERβ. These positive interactions can also be predicted by the combined “ED/VTL approach” (Table S3). The group of fluorotelomer alcohols is represented by 6:2 FTOH and 8:2 FTOH. The Comptox database lists both compounds as inactive towards AR, ERα, and ERβ, while the available literature provides evidence that both react with ERα and that 6:2 FTOH also reacts with ERβ [34,49]. However, if their reactivity with AR, ERα, and ERβ is evaluated with the “ED/VTL approach” (Table S3), both fluorotelomer alcohols are classified as positive for all receptors. Although there is clearly a discrepancy between the predicted interactions with fluorotelomer alcohols for AR and ERβ and the reported in vitro results, it is better to overestimate their endocrine disrupting potential in in silico screening and omit their effects during subsequent in vitro testing than to falsely predict them as inactive at tier 1 and not test them further at all.

Since N-alkyl perfluoroalkyl sulfonamido carboxylates, perfluoroalkyl ether carboxylates, fluorotelomer phosphate esters, fluorotelomer carboxylates, and perfluoroalkyl polyether carboxylates have hardly been studied in vitro, their evaluation with ED and VTL is welcome as an initial screening of their endocrine disrupting effects. The group of N-alkyl perfluoroalkyl sulfonamido-carboxylates can be divided into the sulfonamido-acetic acids NMeFOSSA and NEtFOSSA and the fluorotelomer sulfonic acids (FTS) 4:2 FTS, 6:2 FTS, and 8:2 FTS. The “ED/VTL approach” predicts that both NMeFOSSA and NEtFOSSA react with AR, ERα, and ERβ, while all FTSs react with AR and 8:2 FTS also reacts with both ER isoforms. In the group of fluorotelomer phosphate esters (monoPAPs), the negative predictions of 8:2 monoPAP with the “ED/VTL approach” for AR interactions are confirmed by in vitro data [48,50]. On the other hand, both 6:2 monoPAP and 8:2 monoPAP are predicted to react with both ER isoforms. The 5:3 acid, a representative of the fluorotelomer carboxylates, was found to be active towards AR and ERβ. Finally, the compounds that belong to perfluoroalkyl ether carboxylates and perfluoroalkyl polyether carboxylates are predicted to be inactive towards AR, ERα, and ERβ using the “ED/VTL approach.”

## 4. Discussion

In silico tools are nowadays indispensable in the study of endocrine disruption caused by various chemicals [25]. They can include either QSAR modelling, molecular dynamics simulations, or docking approaches to predict ligand binding to hormone receptors and therefore can serve as a tier 1 screening tool for the assessment of endocrine disrupting effects [43]. Since they mostly predict the compound binding to the selected site of the target, they do not consider activation of feedback loops, reactivity with several targets, bioavailability, unspecific binding, etc. Therefore, in silico results need to be further supported by in vitro and in vivo studies [25].

In training, the models are created with known and specific ligands [51] and later used to test a variety of structurally diverse compounds. In particular, ED has been used to assess the endocrine potential of bisphenols [40], medical drugs [38], PFASs [52], and polyhydroxyalkanoates [42], and VTL has been used to assess organic water contaminants (e.g., nitrosamines, flame retardants, insecticides) [41] and cosmetic ingredients (e.g., triclosan, climbazole) [39]. Depending on the model and its training and modelling approach, the results of the in silico analysis may vary [43], and it is essential that the model is proven to be reliable for the assessment of the specific class of chemicals tested. It has also been claimed that a combination of models leads to more accurate results [31]. In this study, we aimed to investigate the suitability of ED and VTL for assessing the reactivity of PFASs with nuclear receptors.

### 4.1. Suitability of ED and VTL for the Identification of AR Binders

When assessing PFAS reactivity with AR, ED suffered from negative results being misclassified as positive, resulting in a low PPV (50%) and Sp (22%). However, if the ED is used as a tier 1 screening tool, it is better to classify negative results as positive and subject them to in vitro analysis than to classify actual positive compounds as negative and not analyse them at all. However, due to the high number of FPs, ED lacks the ability to reduce the number of chemicals to be tested towards AR. Compared to the predictions of ED for the evaluation of biocidal substances for interaction with AR, where Sp (7%) and MCC (0.08) [31] were low, its predictions for PFASs are more reliable. Lower Se (40%), PPV (36%), and MCC (0.18) were also found in the assessment for identifying AR binding of compounds listed in the US EPA tier 1 dataset [43]. In contrast, VTL provided more balanced results for PFAS interactions with AR. Although a false negative result was obtained compared to ED, there were fewer FPs; thus, the PPV, Sp, Acc, and MCC were improved. In particular, the Acc of ≥75% and MCC of 0.5 indicate a balanced distribution of positive and negative hits, and although some FN results may occur, the vast majority of results are expected to be correct. The use of the combined approaches did not improve the prediction parameters for AR compared to VTL. Therefore, to obtain the most reliable results for identifying the reactivity of PFASs with AR, the use of VTL is recommended.

### 4.2. Suitability of ED and VTL for the Identification of ER Binders

Both ED and VTL were very good at identifying inactive PFASs versus ERα, although positive results can easily be categorised as FNs. Positive results are therefore likely to be TPs, while not all negative results are reliably negative. This observation suggests that screening PFAS reactivity with ERα using ED or VTL could easily result in the loss of actual positive compounds and are therefore not recommended to be used for this purpose. When comparing the parameters for the evaluation of biocidal substances, ED showed comparable Se for PFASs (67% vs. 70% for biocides) but higher Sp (100% vs. 48%) and a higher MCC (0.6 vs. 0.19). However, it should be noted that in our study, only the compounds categorised by ED as belonging to the “red” and “orange” classes were classified as true positives, while Stanojević et al. counted all results belonging to the “yellow,” “orange,” and “red” classes as positive hits [31]. On the other hand, VTL predicted lower Se for PFASs than for the biocides (25% vs. 51%) but higher Sp (100% vs. 67%) and a higher MCC (0.4 vs. 0.16) [31]. Nevertheless, the predictions for ERα could be significantly improved with the positive consensus approach of ED and VTL, where the proportion of false negatives was greatly reduced, resulting in an Acc of 93% and an MCC of 0.9.

In the case of ERβ, both ED and VTL tended to confound positive and negative results, resulting in poor prediction parameters (PPV and NPV 50–71%), especially in the case of VTL, with an MCC of 0.1. However, compared to their suitability for the evaluation of biocidal compounds, the prediction parameters were comparable or even better. In particular, ED provided poorer predictions for the biocides, as assessed by Se (11% for biocides vs. 67% for PFAS), Sp (0.29% vs. 71%), and MCC (−0.03 vs. 0.4) [31]. For VTL, Se was lower for PFASs (51% for biocides and 33% for PFAS), while Sp was better (0.67% vs. 75%) and MCC was comparable (0.17 vs. 0.1) [31]. Predictions for ERβ were improved by the positive consensus model, in which the number of FNs was reduced, leading to an improvement in Acc (71%) and MCC (0.5). The combination of models using the negative consensus approach is not recommended, as it led to a high proportion of FNs and a reduction in MCC (−0.1).

### 4.3. Suitability of ED and VTL for the Identification of GR Binders

Our literature search revealed no data confirming that PFASs have a measurable effect on GR modulation. Moreover, a previously published study by Singam et al. revealed that only 9 of over 4000 PFASs tested were predicted to be GR antagonists, and none of them were predicted to be its agonists [53]. In accordance with the literature data, ED categorised all interactions between PFASs and GR as negative. However, in the absence of positive results from in vitro tests, we cannot judge whether it would correctly identify potential PFASs with glucocorticoid activity. VTL, on the other hand, incorrectly predicted most PFASs as reactive to GR, resulting in a low Sp and Acc (18%). Due to the lack of actual positive hits, we were unable assess the suitability of ED and VTL for identifying GR-positive hits, but ED performed better than VTL in the identification of negative results.

### 4.4. Suitability of ED and VTL for the Identification of PPAR Binders

With regard to PPARα and PPARγ, ED correctly classified non-active PFASs as negative but also recognised all PFASs active in in vitro tests as negative. However, it should be noted that although there are several data showing that PFASs reacted with PPARs (see Table S4), their active concentrations usually exceeded 100 µM, which is above the in vivo and toxicologically relevant concentrations [32,54,55,56,57]. In accordance with the literature data, in silico models developed by Kowalska et al. also predicted that PFASs bind to PPARα, β, and γ with low or moderate probability [58]. Therefore, their negative placement by ED could still represent their potency in vivo. VTL showed better identification of actual positives in terms of PPARγ than ED, although the model appears to be promiscuous enough to identify true negatives as FPs. Judging by the PPV of 62% and Se of 89%, the majority of actual positives are likely to have been correctly identified, but the low NPV (67%) and Sp (29%) indicate problems with actual negatives. Furthermore, the MCC of 0.1 suggests that the predictions were close to random. Even when using combinations of both models, the prediction parameters were not improved. Therefore, we cannot recommend the use of VTL for the prediction of the interaction of PFASs with PPARγ.

### 4.5. Assessment of PFAS Reactivity with Nuclear Receptors and Preliminary Structure–Activity Relationship

Based on the prediction parameters determined in this study, VTL was identified as a suitable tool for predicting PFAS interactions with AR and a positive consensus of ED and VTL for PFAS interactions with ERα and ERβ. Following the “ED/VTL approach” in combination with the available in vitro data, this study provides evidence that the lipophilicity of perfluoroalkane sulfonates correlates with an increased potential for reactivity with AR and ERs. Similar observations have been also reported in studies where PFASs have been assayed by ED [59] or other in silico tools [52] and have also been confirmed in vitro. The long-chain and thus more lipophilic PFASs were found to be more toxic than the short-chain compounds, as shown on cultured ovarian follicles, where exposure to long-chain PFASs showed poorer gonadotropin-dependent follicle growth, ovulation, and hormone secretion in comparison with control follicles [60]. On mouse liver organoids, short-chain PFASs caused less pronounced cytomorphological changes and did not activate apoptosis, unlike long-chain PFOS and PFOA [61]. A trend of the logP relationship to nuclear receptor targeting was also predicted by the “ED/VTL approach” for perfluoroalkyl carboxylates, although the in vitro studies did not confirm the reactivity of the most lipophilic PFUnA with AR and ERβ or PFDA with ERβ. Through a regulatory perspective, identification of negative results as positives in the tier 1 in silico screening is not controversial, since it is better to omit the predictions in tier 2 or tier 3 than falsely predict their inactivity and not subject them to wet lab assays at all.

The major limitation of this study is the small number of studies available to evaluate the results predicted by ED and VTL. Our literature search of 33 compounds found only 16 PFASs for which data were available on their reactivity with at least one nuclear receptor. Therefore, we used the “ED/VTL approach” to identify the potential reactivity of under-studied PFAS compounds. In the group of perfluoroalkyl carboxylates, no in vitro data on PFMOPrA were found, while the “ED/VTL approach” identified it to be inactive towards the investigated receptors. Regarding the perfluoroalkane sulfonates, the in silico analysis found PFHpS, PFNS, and PFDS to interact with AR, PFNS, PFDS, and 9-Cl with ERα, and found PFHpS, PFNS, PFDS, and 9-Cl to interact with ERβ. The “ED/VTL” approach also showed that the 6:2 and 8:2 monoPAPs react with ERβ and that 8:2 monoPAP also reacted with ERα, while the 5:3 acid was active towards AR and ERβ. The group of N-alkyl perfluoroalkylsulfonamido carboxylates was proven to be reactive towards AR and ERβ, with the exception of 4:2 FTS and 6:2 FTS, which were found to be inactive towards ERβ. On the other hand, perfluoroalkyl (poly)ether carboxylates were found to be inactive towards all receptors analysed.

## 5. Conclusions

This comparative in silico study identified VTL as a suitable tool for the identification of PFAS targeting ARs, and the positive consensus approach of ED and VTL for the identification of PFASs targeting ERα and ERβ. These models can be used as a tier 1 screening approach for assessing the endocrine potential of PFASs, with the expectation that the negative hits are non-reactive and reliably do not interact with ARs, ERα, or ERβ. Using ED and VTL, PFHpS, PFNS, PFDS, 9-Cl, NMeFOSAA, NEt-FOSAA, 4:2 FTS, 6:2 FTS, 8:2 FTS, 6:2 monoPAP, 8:2 monoPAP, and 5:3 acid were identified as potential ligands for ARs and/or ERs in this study. Since no in vitro data are available to support these results, the PFASs mentioned remain be investigated in the wet lab experiments to close the data gaps regarding their safety.

## Figures and Tables

**Table 1:** Results of selected PFAS reactivity with nuclear receptors as predicted by ED or VTL or determined in in vitro studies.

		Perfluoroalkyl Carboxylates								Perfluoroalkane Sulfonates			Perfluoroalkane Sulfonamides	Fluorotelomer Alcohols		Perfluoroalkyl Ether Carboxylates	Fluorotelomer Phosphate Esters
R	Model	PFBA	PFPeA	PFHxA	PFHpA	PFOA	PFNA	PFDA	PFUnA	PFBS	PFHxS	PFOS	PFOSA	6:2 FTOH	8:2 FTOH	HFPO-DA	8:2 monoPAP
ARs	ED	−6.90	−7.30	−7.60	−9.00	−8.90	−9.40	−9.70	−10.40	−7.90	−8.60	−9.70	−9.80	−8.60	−9.40	−8.00	−9.10
	ED an.	−6.80	−7.30	−8.20	−8.70	−8.80	−9.20	−9.60	−10.20	−8.20	−8.30	−9.70	−9.50	−8.50	−9.47	−7.90	−9.10
	VTL	>100	>100	>100	>100	31.70	33.20	17.40	8.80	>100	97.60	60.00	15.40	4.32	0.62	>100	>100
	in vitro	N	N	N	P	P	P	P	N	N	P	P	P	N	N	N	N
ERα	ED	−5.70	−6.40	−7.10	−7.70	−8.50	−9.00	−9.30	−9.80	−6.80	−8.10	−9.10	−9.40	−8.00	−9.00	−7.10	−9.20
	ED an.	−5.90	−6.50	−7.00	−7.80	−8.50	−9.20	−9.60	−9.90	−6.70	−8.10	−9.20	−9.30	−8.00	−8.90	−7.00	−8.70
	VTL	>100	>100	>100	>100	>100	>100	>100	>100	>100	>100	>100	>100	5.58	1.51	>100	>100
	in vitro	N	/	N	N	P	P	P	P	N	N	P	P	P	P	N	/
ERβ	ED	−6.00	−6.80	−7.50	−8.20	−9.30	−9.70	−9.80	−10.40	−7.20	−8.70	−9.70	−9.90	−8.40	−9.03	−7.90	−9.40
	ED an.	−5.80	−6.60	−7.30	−7.90	−8.50	−9.20	−9.70	−9.70	−7.00	−8.30	−9.40	−9.40	−7.60	−9.10	−7.20	−8.80
	VTL	>100	>100	>100	>100	>100	>100	>100	96.20	>100	>100	>100	93.70	4.30	0.71	>100	>100
	in vitro	N	/	N	P	P	P	N	N	N	N	P	P	P	N	N	/
GRs	ED	−5.60	−5.90	−6.80	−7.30	−7.60	−8.40	−9.20	−9.60	−6.40	−7.50	−8.50	−8.60	−7.40	−8.13	−7.00	−9.10
	ED an.	−5.60	−6.10	−6.40	−6.80	−7.50	−7.90	−7.90	−8.20	−6.50	−7.00	−7.80	−7.80	−7.20	−7.97	−6.50	−7.70
	VTL	>100	85.50	23.70	>100	14.10	24.00	32.40	6.32	>100	8.40	56.40	42.00	18.60	4.54	>100	53.50
	in vitro	/	/	N	N	N	N	N	N	/	/	N	N	N	N	N	/
PPARα	ED	−5.60	−6.30	−7.10	−7.50	−8.00	−8.10	−8.50	−8.80	−6.30	−7.20	−8.10	−8.30	−7.30	−8.30	−6.70	−8.40
	in vitro	N	P	P	P	P	P	P	P	P	P	P	N	N	N	P	N
PPARγ	ED	−6.10	−6.60	−7.20	−7.80	−7.90	−8.30	−8.90	−9.40	−6.90	−7.40	−8.70	−8.50	−7.80	−8.50	−7.30	−8.70
	VTL	>100	>100	27.10	26.30	33.00	30.20	6.16	6.44	91.90	46.00	1.77	1.74	35.50	11.20	45.70	>100
	in vitro	N	P	P	P	P	P	P	N	N	P	P	N	N	N	P	N

R—receptor; ED—Endocrine Disruptome; ED an.—Endocrine Disruptome antagonistic mode; VTL—VirtualToxLab; P—positive in vitro result; N—negative in vitro result; /—no in vitro data available. Results in green, yellow, orange, and red cells represent results are outputs of ED in kcal/mol, where red colour represents high binding probability, orange represents moderate binding probability, yellow mild binding probability, and green low binding probability. Results in white cells are outputs of VTL in µM where the values > 100 µM are considered “not binding.”

**Table 2:** Evaluation of ED with in vitro data.

	TP	FP	TN	FN	PPV [%]	Se [%]	NPV [%]	Sp [%]	Acc [%]	MCC
AR	7	7	2	0	50	100	100	22	56	0.3
ERα	6	0	6	2	100	75	75	100	86	0.8
ERβ	4	2	5	2	67	67	71	71	69	0.4
GR	0	0	11	0			100	100	100	
PPARα	0	0	5	11		0	31	100	31	
PPARγ	0	0	7	9		0	44	100	44	

AR—androgen receptor; ER—oestrogen receptor; GR—glucocorticoid receptor; PPAR—peroxisome proliferator-activated receptor; TP—true positive; FP—false positive; TN—true negative; FN—false negative; PPV—positive prediction value; Se—sensitivity; NPV—negative prediction value; Sp—specificity; Acc—accuracy; MCC—Matthews correlation coefficient.

**Table 3:** Evaluation of VTL with in vitro data.

	TP	FP	TN	FN	PPV [%]	Se [%]	NPV [%]	Sp [%]	Acc [%]	MCC
AR	6	3	6	1	67	86	86	67	75	0.5
ERα	2	0	6	6	100	25	50	100	57	0.4
ERβ	2	2	6	4	50	33	60	75	57	0.1
GR	0	9	2	0	0		100	18	18	
PPARγ	8	5	2	1	62	89	67	29	63	0.2

AR—androgen receptor; ER—oestrogen receptor; GR—glucocorticoid receptor; PPAR—peroxisome proliferator-activated receptor; TP—true positive; FP—false positive; TN—true negative; FN—false negative; PPV—positive prediction value; Se—sensitivity; NPV—negative prediction value; Sp—specificity; Acc—accuracy; MCC—Matthews correlation coefficient.

**Table 4:** Positive consensus model.

	TP	FP	TN	FN	PPV [%]	Se [%]	NPV [%]	Sp [%]	Acc [%]	MCC
AR	7	7	2	0	50	100	100	22	56	0.3
ERα	7	0	6	1	100	88	86	100	93	0.9
ERβ	5	3	5	1	63	83	83	63	71	0.5
GR	0	8	3	0	0		100	27	27	
PPARγ	8	5	2	1	62	89	67	29	63	0.2

AR—androgen receptor; ER—oestrogen receptor; GR—glucocorticoid receptor; PPAR—peroxisome proliferator-activated receptor; TP—true positive; FP—false positive; TN—true negative; FN—false negative; PPV—positive prediction value; Se—sensitivity; NPV—negative prediction value; Sp—specificity; Acc—accuracy; MCC—Matthews correlation coefficient.

**Table 5:** Negative consensus model.

	TP	FP	TN	FN	PPV	Se [%]	NPV [%]	Sp [%]	Acc [%]	MCC
AR	6	3	6	1	67	86	86	67	75	0.5
ERα	1	0	6	7		17	50	100	55	0.3
ERβ	1	2	6	5	33	17	55	75	50	−0.1
GR	0	0	11	0			100	100	100	
PPARγ	0	0	7	9		0	44	100	44	

AR—androgen receptor; ER—oestrogen receptor; GR—glucocorticoid receptor; PPAR—peroxisome proliferator-activated receptor; TP—true positive; FP—false positive; TN—true negative; FN—false negative; PPV—positive prediction value; Se—sensitivity; NPV—negative prediction value; Sp—specificity; Acc—accuracy; MCC—Matthews correlation coefficient.

**Table 6:** Interactions of PFASs with AR, ERα, and ERβ as predicted by the “ED/VTL” approach or determined in the in vitro studies.

	Perfluoroalkyl Carboxylates									Perfluoroalkane Sulfonates								Perfluoroalkane Sulfonamides	N-Alkyl Perfluoroalkyl Sulfonamido Carboxylates					Fluorotelomer Alcohols		Perfluoroalkyl Ether Carboxylates				Fluorotelomer Phosphate Esters		Fluorotelomer Carboxylates	Perfluoroalkyl Polyether Carboxylates
	**PFMOPrA**	**PFBA**	**PFPeA**	**PFHxA**	**PFHpA**	**PFOA**	**PFNA**	**PFDA**	**PFUnA**	**PFBS**	**PFPeS**	**PFHxS**	**PFHpS**	**PFOS**	**PFNS**	**PFDS**	**9-Cl**	**PFOSA**	**NMeFOSAA**	**NEtFOSAA**	**4:2 FTS**	**6:2 FTS**	**8:2 FTS**	**6:2 FTOH**	**8:2 FTOH**	**HFPO-DA**	**ADONA**	**PFMBA**	**PFEESA**	**6:2 monoPAP**	**8:2 monoPAP**	**5:3 Acid**	**PFECA B**
AR	*N*	**N**	**N**	**N**	**P**	**P**	**P**	**P**	**N**	**N**	*N*	**P**	*P*	**P**	*P*	*P*	*N*	**P**	*P*	*P*	*P*	*P*	*P*	**N**	**N**	**N**	*N*	*N*	*N*	*N*	**N**	*P*	*N*
ERα	*N*	**N**	*N*	**N**	**N**	**P**	**P**	**P**	**P**	**N**	*N*	**N**	*N*	**P**	*P*	*P*	*P*	**P**	*P*	*P*	*N*	*N*	*P*	**P**	**P**	**N**	*N*	*N*	*N*	*N*	*P*	*N*	*N*
ERβ	*N*	**N**	*N*	**N**	**P**	**P**	**P**	**N**	**N**	**N**	*N*	**N**	*P*	**P**	*P*	*P*	*P*	**P**	*P*	*P*	*N*	*N*	*P*	**P**	**P**	**N**	*N*	*N*	*N*	*P*	*P*	*P*	*N*
#	0	0	0	0	2	3	3	2	1	0	0	1	1	3	3	3	2	3	3	3	1	1	3	2	2	0	0	0	0	1	2	2	0

P—positive result; N—negative result; #—number of positive results. Results in bold are derived from in vitro studies. Results in italics are derived from the “ED/VTL approach.”

## Data Availability

The original contributions presented in this study are included in the article/supplementary material. Further inquiries can be directed to the corresponding author(s).

## Supplementary Materials

The following supporting information can be downloaded at: https://www.mdpi.com/article/10.3390/jox15050136/s1, Table S1: PFASs selected for this study; Table S2: The threshold values of binding energies (kcal × mol^−1^) for classification of compounds within high (red), moderate (orange), mild (yellow), or low (green) probability of binding with Endocrine Disruptome; Table S3: Complete results of analysis with ED and VTL; Table S4: Summary of in vitro studies used for evaluation of ED and VTL. References [29,32,33,34,35,47,48,49,50,54,55,56,57,62,63,64,65,66,67,68,69,70,71,72,73,74,75] are cited in the Supplementary material and/or in the main text.

## Abbreviations

The following abbreviations are used in this manuscript:

PFMOPrA	Perfluoro-3-methoxypropanoic acid
PFBA	Perfluorobutanoic acid
PFPeA	Perfluoropentanoic acid
PFHxA	Perfluorohexanoic acid
PFHpA	Perfluoroheptanoic acid
PFOA	Perfluorooctanoic acid
PFNA	Perfluorononanoic acid
PFDA	Perfluorodecanoic acid
PFUnA	Perfluoroundecanoic acid
PFBS	Perfluorobutanesulfonic acid
PFPeS	Perfluoropentanesulfonic acid
PFHxS	Perfluorohexanesulfonic acid
PFHpS	Perfluoroheptanesulfonic acid
PFOS	Perfluorooctanesulfonic acid
PFNS	Perfluorononanesulfonic acid
PFDS	Perfluorodecanesulfonic acid
9-Cl	9-Chlorohexadecafluoro-3-oxanonane-1-sulfonic acid
PFOSA	Perfluorooctanesulfonamide
NMeFOSAA	2-(N-Methylperfluorooctanesulfonamido)acetic acid
NEtFOSAA	2-(N-Ethylperfluorooctanesulfonamido)acetic acid
4:2 FTS	4:2 Fluorotelomer sulfonic acid
6:2 FTS	6:2 Fluorotelomer sulfonic acid
8:2 FTS	8:2 Fluorotelomer sulfonic acid
8:2 FTOH	8:2 Fluorotelomer alcohol
6:2 FTOH	6:2 Fluorotelomer alcohol
HFPO-DA	Perfluoro-2-methyl-3-oxahexanoic acid
ADONA	4,8-Dioxa-3H-perfluorononanoic acid
PFMBA	Perfluoro(4-methoxybutanoic) acid
PFEESA	Perfluoro(2-ethoxyethane)sulfonic acid
8:2 monoPAP	8:2 Fluorotelomer dihydrogen phosphate
6:2 monoPAP	6:2 Fluorotelomer phosphate monoester
5:3 acid	2H,2H,3H,3H-perfluorooctanoic acid
PFECA B	Perfluoro-3,6-dioxaheptanoic acid
