# Supplementary material for: Evaluation of Endocrine Disruptome and VirtualToxLab for Predicting Per- and Polyfluoroalkyl Substances Binding to Nuclear Receptors
Source: J Xenobiot. 2025 Aug 22;15(5):136. doi: 10.3390/jox15050136 (PMC12452740; doi:10.3390/jox15050136)
Supplement: Supplementary file 1 [file jox-15-00136-s001.zip › jox-3769612-supplementary.pdf]

Journal of Xenobiotics

MDPI

Evaluation of Endocrine Disruptome and VirtualToxLab for Predicting Per- and Polyfluoroalkyl Substances Binding to Nuclear Receptors

Nina Franko, Manca Vetrih and Marija Sollner Dolenc

Table S1: PFAS selected for this study.

	name	CAS	MW	logP	molecular formula	SMILES
**Perfluoroalkyl carboxylates**						
1	PFMOPrA	377-73-1	230,04	2,3	$CF_3OCF_2CF_2COOH$	C(=O)(C(C(OC(F)(F)F)(F)F)(F)F)O
2	PFBA	375-22-4	214,04	2,2	C_3F_7COOH	C(=O)(C(C(C(F)(F)F)(F)F)(F)F)O
3	PFPeA	2706-90-3	264,05	2,9	C_4F_9COOH	C(=O)(C(C(C(C(F)(F)F)(F)F)(F)F)(F)F)O
4	PFHxA	307-24-4	314,05	3,6	$C_5F_{11}COOH$	C(=O)(C(C(C(C(C(F)(F)F)(F)F)(F)F)(F)F)(F)F)O
5	PFHpA	375-85-9	364,06	4,3	$C_6F_{13}COOH$	C(=O)(C(C(C(C(C(C(F)(F)F)(F)F)(F)F)(F)F)(F)F)(F)F)O
6	PFOA	335-67-1	414,07	4,9	$C_7F_{15}COOH$	C(=O)(C(C(C(C(C(C(C(F)(F)F)(F)F)(F)F)(F)F)(F)F)(F)F)(F)F)O
7	PFNA	375-95-1	464,08	5,6	$C_8F_{17}COOH$	C(=O)(C(C(C(C(C(C(C(C(F)(F)F)(F)F)(F)F)(F)F)(F)F)(F)F)(F)F)(F)F)O
8	PFDA	335-76-2	514,08	6,3	$C_9F_{19}COOH$	C(=O)(C(C(C(C(C(C(C(C(C(F)(F)F)(F)F)(F)F)(F)F)(F)F)(F)F)(F)F)(F)F)(F)F)O
9	PFUnA	2058-94-8	564,09	6,9	$C_{10}F_{21}COOH$	C(=O)(C(C(C(C(C(C(C(C(C(C(F)(F)F)(F)F)(F)F)(F)F)(F)F)(F)F)(F)F)(F)F)(F)F)(F)F)O
**Perfluoroalkane sulfonates**						
10	PFBS	375-73-5	300,1	2,3	$C_4F_9SO_2OH$	C(C(C(F)(F)S(=O)(=O)O)(F)F)(C(F)(F)F)(F)F
11	PFPeS	2706-91-4	350,11	3	$C_5F_{11}SO_2OH$	C(C(C(F)(F)F)(F)F)(C(C(F)(F)S(=O)(=O)O)(F)F)(F)F
12	PFHxS	355-46-4	400,12	3,7	$C_6F_{13}SO_2OH$	C(C(C(C(F)(F)S(=O)(=O)O)(F)F)(F)F)(C(C(F)(F)F)(F)F)(F)F
13	PFHpS	375-92-8	450,12	4,3	$C_7F_{15}SO_2OH$	C(C(C(C(F)(F)F)(F)F)(F)F)(C(C(C(F)(F)S(=O)(=O)O)(F)F)(F)F)(F)F
14	PFOS	1763-23-1	500,13	5	$C_8F_{17}SO_2OH$	C(C(C(C(C(F)(F)S(=O)(=O)O)(F)F)(F)F)(F)F)(C(C(C(F)(F)F)(F)F)(F)F)(F)F
15	PFNS	68259-12-1	550,14	5,7	$C_9F_{19}SO_2OH$	C(C(C(C(C(F)(F)F)(F)F)(F)F)(F)F)(C(C(C(C(F)(F)S(=O)(=O)O)(F)F)(F)F)(F)F)(F)F

16	PFDS	335-77-3	600,15	6,4	$C_{10}F_{21}SO_2OH$	C(C(C(C(C(C(F)(F)S(=O)(=O)O)(F)F)(F)F)(F)F)(F)F)(C(C(C(C(F)(F)F)(F)F)(F)F)(F)F)(F)F
17	9-Cl	756426-58-1	532,58	5,3	$C_8HClF_{16}O_4S$	C(C(C(C(F)(F)Cl)(F)F)(F)F)(C(C(OC(C(F)(F)S(=O)(=O)O)(F)F)(F)F)(F)F)(F)F
**Perfluoroalkane sulfonamides**						
18	PFOSA	754-91-6	499,15	4,8	$C_8F_{17}SO_2NH_2$	C(C(C(C(C(F)(F)S(=O)(=O)N)(F)F)(F)F)(F)F)(C(C(C(F)(F)F)(F)F)(F)F)(F)F
**N-alkyl perfluoroalkyl sulfonamido carboxylates**						
19	NMeFOSAA	2355-31-9	571,21	5	$C_8F_{17}SO_2N(CH_3)CH_2COOH$	CN(CC(=O)O)S(=O)(=O)C(C(C(C(C(C(C(C(F)(F)F)(F)F)(F)F)(F)F)(F)F)(F)F)(F)F)(F)F
20	NEt-FOSAA	2991-50-6	585,24	5,4	$C_8F_{17}SO_2N(C_2H_5)CH_2COOH$	CCN(CC(=O)O)S(=O)(=O)C(C(C(C(C(C(C(C(F)(F)F)(F)F)(F)F)(F)F)(F)F)(F)F)(F)F)(F)F
21	4:2 FTS	757124-72-4	328,15	2,6	$C_4F_9CH_2CH_2SO_3H$	C(CS(=O)(=O)O)C(C(C(C(F)(F)F)(F)F)(F)F)(F)F
22	8:2 FTS	39108-34-4	528,18	5,3	$C_8F_{17}CH_2CH_2SO_3H$	C(CS(=O)(=O)O)C(C(C(C(C(C(C(C(F)(F)F)(F)F)(F)F)(F)F)(F)F)(F)F)(F)F)(F)F
23	6:2 FTS	27619-97-2	428,17	3,9	$C_6F_{13}CH_2CH_2SO_3H$	C(CS(=O)(=O)O)C(C(C(C(C(C(F)(F)F)(F)F)(F)F)(F)F)(F)F)(F)F
**Fluorotelomer alcohols**						
24	6:2 FTOH	647-42-7	364,1	4,4	$C_6F_{13}CH_2CH_2OH$	C(CO)C(C(C(C(C(C(F)(F)F)(F)F)(F)F)(F)F)(F)F)(F)F
25	8:2 FTOH	678-39-7	464,12	5,7	$C_8F_{17}CH_2CH_2OH$	C(CO)C(C(C(C(C(C(C(C(F)(F)F)(F)F)(F)F)(F)F)(F)F)(F)F)(F)F)(F)F
**Perfluoroalkyl ether carboxylates**						
26	HFPO-DA	13252-13-6	330,05	3,6	$C_3F_7OCF(CF_3)COOH$	C(=O)(C(C(F)(F)F)(OC(C(C(F)(F)F)(F)F)(F)F)F)O
27	ADONA	919005-14-4	378,07	4,1	$CF_3OCF_2CF_2CF_2OCFHCF_2COOH$	C(C(C(=O)O)(F)F)(OC(C(C(OC(F)(F)F)(F)F)(F)F)(F)F)F
28	PFMBA	863090-89-5	280,04	3	$C_5HF_9O_3$	C(=O)(C(C(C(OC(F)(F)F)(F)F)(F)F)(F)F)O
29	PFEESA	113507-82-7	316,1	2,1	$C_4HF_9H_4S$	C(C(F)(F)F)(OC(C(F)(F)S(=O)(=O)O)(F)F)(F)F
**Fluorotelomer phosphate esters**						
30	8:2 monoPAP	57678-03-2	544,1	4,6	$C_{10}H_6F_{17}O_4P$	C(COP(=O)(O)O)C(C(C(C(C(C(C(C(F)(F)F)(F)F)(F)F)(F)F)(F)F)(F)F)(F)F)(F)F
31	6:2 monoPAP	57678-01-0	444,08	3,3	$C_6F_{13}CH_2CH_2OP(=O)(OH)_2$	C(COP(=O)(O)O)C(C(C(C(C(C(F)(F)F)(F)F)(F)F)(F)F)(F)F)(F)F
**Fluorotelomer carboxylates**						
32	5:3 acid	914637-49-3	342,11	3,9	$C_8H_5F_{11}O_2$	C(CC(C(C(C(C(F)(F)F)(F)F)(F)F)(F)F)(F)F)C(=O)O
**Perfluoroalkyl polyether carboxylates**						

33	PFECA B	151772-58-6	296,04	3	$C_5HF_9O_4$	C(=O)(C(OC(C(OC(F)(F)F)(F)F)(F)F)(F)F)O

Table S2: The threshold values of binding energies (kcal x mol-1) for classification of compounds within high (red), moderate (orange), mild (yellow) or low (green) probability of binding with Endocrine Disruptome [1].

probability of interaction / receptor	AR	AR an.	ER α	ER α an.	ER β	ER β an.	GR	GR an.	LXRα	LXRβ	MR	PPARα	PPARβ	PPARγ	RXRα	TRα	TRβ
high	> - 8.6	> - 8.4	> - 9.3	> - 10.7	> - 9.2	> - 9.0	> - 10.7	> - 9.8	> - 11.9	> - 12.1	> - 8.5	> - 10.0	> - 10.5	> - 10.3	> - 12.1	> - 10.2	> - 10.5
moderate	> - 8.1	> - 7.6	> - 8.8	> - 9.5	> - 8.7	> - 8.6	> - 9.7	> - 9.2	> - 10.7	> - 11.3	> - 8.2	> - 9.4	> - 10.1	> - 9.6	> - 10.8	> - 9.2	> - 9.4
mild	> - 7.4	> - 3.1	> - 8.2	> - 8.6	> - 8.0	> - 8.3	> - 7.3	> - 8.5	> - 9.8	> - 10.3	> - 6.9	> - 8.9	> - 9.6	> - 8.9	> - 10.0	> - 7.2	> - 7.8
low	< - 7.4	< - 3.1	< - 8.2	< - 8.6	< - 8.0	< - 8.3	< - 7.3	< - 8.5	< - 9.8	< - 10.3	< - 6.9	< - 8.9	< - 9.6	< - 8.9	< - 10.0	< - 7.2	< - 7.8

Legend: an. - antagonistic mode

Table S3: Complete results of analysis with ED and VTL.

	Perfluoroalkyl carboxylates									Perfluoroalkane sulfonates							
receptor/ PFAS	PFMOPrA	PFBA	PFPeA	PFHxA	PFHpA	PFOA	PFNA	PFDA	PFUnA	PFBS	PFPeS	PFHxS	PFHpS	PFOS	PFNS	PFDS	9-Cl
AR	-6,73	-6,90	-7,30	-7,60	-9,00	-8,90	-9,40	-9,70	-10,40	-7,90	-8,50	-8,60	-9,20	-9,70	-10,20	-9,70	-9,60
AR an.	-6,40	-6,80	-7,30	-8,20	-8,70	-8,80	-9,20	-9,60	-10,20	-8,20	-8,50	-8,30	-9,10	-9,70	-9,90	-9,70	-9,50
AR VTL	>100	>100	>100	>100	>100	31,70	33,20	17,40	8,80	>100	>100	97,60	69,00	60,00	7,11	2,48	>100
ER α	-6,03	-5,70	-6,40	-7,10	-7,70	-8,50	-9,00	-9,30	-9,80	-6,80	-7,40	-8,10	-8,60	-9,10	-9,70	-10,10	-9,00
ER a an.	-5,80	-5,90	-6,50	-7,00	-7,80	-8,50	-9,20	-9,60	-9,90	-6,70	-7,50	-8,10	-8,60	-9,20	-9,50	-9,80	-8,90
ER α VTL	>100	>100	>100	>100	>100	>100	>100	>100	>100	>100	>100	>100	>100	>100	>100	>100	>100
ER β	-5,90	-6,00	-6,80	-7,50	-8,20	-9,30	-9,70	-9,80	-10,40	-7,20	-7,90	-8,70	-9,40	-9,70	-10,10	-10,10	-9,20
ER β an.	-5,60	-5,80	-6,60	-7,30	-7,90	-8,50	-9,20	-9,70	-9,70	-7,00	-7,40	-8,30	-9,00	-9,40	-9,30	-9,30	-9,25
ER β VTL	>100	>100	>100	>100	>100	>100	>100	>100	96,20	>100	>100	>100	>100	>100	>100	>100	25,90
GR	-5,83	-5,60	-5,90	-6,80	-7,30	-7,60	-8,40	-9,20	-9,60	-6,40	-7,30	-7,50	-7,70	-8,50	-9,30	-9,80	-8,50
GR an	-6,03	-5,60	-6,10	-6,40	-6,80	-7,50	-7,90	-7,90	-8,20	-6,50	-6,50	-7,00	-7,40	-7,80	-8,00	-8,40	-7,60
GR VTL	>100	>100	85,50	23,70	>100	14,10	24,00	32,40	6,32	>100	26,00	8,40	>100	56,40	3,43	1,32	24,70
PPAR α	-5,60	-5,60	-6,30	-7,10	-7,50	-8,00	-8,10	-8,50	-8,80	-6,30	-6,90	-7,20	-8,00	-8,10	-8,70	-8,90	-8,50
PPAR β	-6,30	-6,40	-7,10	-7,30	-8,20	-8,40	-9,10	-9,90	-9,70	-7,20	-7,70	-8,10	-8,50	-9,20	-9,70	-10,10	-9,30
PPAR y	-6,07	-6,10	-6,60	-7,20	-7,80	-7,90	-8,30	-8,90	-9,40	-6,90	-7,40	-7,40	-8,10	-8,70	-8,80	-9,30	-9,10
PPAR y VTL	>100	>100	>100	27,10	26,30	33,00	30,20	6,16	6,44	91,90	22,10	46,00	19,20	1,77	5,69	3,76	7,56

	Perfluoro alkane sulfonamides	N-alkyl perfluoroalkyl sulfonamido carboxylates					Fluorotelomer alcohols		Perfluoroalkyl ether carboxylates				Fluorotelomer phosphate esters		Fluorotelomer carboxylates	Perfluoroalkyl polyether carboxylates
receptor/ PFAS	PFOSA	NMeFOSAA	NEtFOSAA	4:2 FTS	6:2 FTS	8:2 FTS	6:2 FTOH	8:2 FTOH	HFPO-DA	ADONA	PFMBA	PFEESA	6:2 monoP	8:2 monoPAP	5:3 acid	PFECA B
AR	-9,80	-9,00	-8,50	-7,80	-8,77	-9,30	-8,60	-9,40	-8,00	-8,00	-7,40	-7,80	-8,57	-9,10	-8,63	-7,77
AR an.	-9,50	-9,10	-8,80	-7,60	-8,53	-9,20	-8,50	-9,47	-7,90	-7,60	-7,40	-7,70	-8,33	-9,10	-8,30	-7,50
AR VTL	15,40	1,87	2,11	37,30	10,40	2,77	4,32	0,62	>100	>100	>100	>100	>100	>100	83,50	>100
ER α	-9,40	-9,10	-8,80	-7,10	-8,03	-9,20	-8,00	-9,00	-7,10	-7,10	-6,30	-6,50	-8,17	-9,20	-7,47	-6,40
ER a an.	-9,30	-8,50	-8,40	-6,80	-8,03	-8,83	-8,00	-8,90	-7,00	-7,40	-6,60	-6,80	-8,30	-8,70	-7,47	-6,80
ER α VTL	>100	52,20	83,50	>100	>100	>100	5,58	1,51	>100	>100	>100	>100	>100	>100	>100	>100
ER β	-9,90	-9,60	-9,00	-7,40	-8,60	-9,60	-8,40	-9,03	-7,90	-7,60	-6,70	-6,90	-8,73	-9,40	-8,07	-6,80
ER β an.	-9,40	-7,90	-7,90	-6,90	-8,30	-8,50	-7,60	-9,10	-7,20	-7,20	-6,50	-6,90	-8,20	-8,80	-7,40	-6,43
ER β VTL	93,70	>100	13,50	>100	>100	>100	4,30	0,71	>100	>100	>100	>100	>100	>100	51,20	>100
GR	-8,60	-9,30	-9,40	-6,80	-8,10	-9,03	-7,40	-8,13	-7,00	-6,90	-6,30	-6,70	-8,07	-9,10	-7,30	-6,53
GR an	-7,80	-7,80	-7,60	-6,50	-6,97	-7,47	-7,20	-7,97	-6,50	-6,60	-6,20	-6,20	-6,93	-7,70	-6,50	-6,40
GR VTL	42,00	0,23	0,36	12,50	47,50	1,71	18,60	4,54	>100	>100	>100	>100	>100	53,50	12,90	>100
PPAR α	-8,30	-8,20	-8,00	-6,80	-7,50	-8,47	-7,30	-8,30	-6,70	-7,10	-6,50	-6,30	-7,50	-8,40	-7,07	-6,40
PPAR β	-9,20	-9,40	-9,30	-7,50	-8,10	-9,23	-7,80	-8,90	-7,20	-7,60	-7,00	-7,00	-8,13	-9,10	-7,87	-7,10
PPAR y	-8,50	-8,60	-8,50	-6,90	-8,00	-8,67	-7,80	-8,50	-7,30	-7,50	-6,90	-6,70	-7,83	-8,70	-7,70	-7,00
PPAR y VTL	1,74	0,59	0,48	4,40	3,85	3,25	35,50	11,20	45,70	27,60	98,20	21,30	>100	>100	14,00	24,70

AR – androgen receptor; ER – estrogen receptor; GR – glucocorticoid receptor; PPAR - Peroxisome Proliferator-Activated Receptor; ED – Endocrine Disruptome; an. – antagonistic mode; VTL – VirtualToxLab. Results in green, yellow, orange and red cells represent results that are outputs of ED in kcal/mol, results in white cells are outputs of VTL in μM.

Table S4: Summary of *in vitro* studies used for evaluation of ED and VTL.

Compound (CAS)	Nuclear receptor	Positive	Negative	CompTox data-base	decision
**PFBS (375-73-5)**	**AR**	ND	No effect [2] Inactive Ar eco screen [3]	ND	inactive
	**ER α**	Binding Kd 131,81 ± 4,58 μM, likely antagonist in silico [4]	No effect [2]	ND	inactive
	**ER β**	ND	No effect [2]	ND	inactive
	**GR**	ND	ND	ND	/
	**PPAR α**	Yes, activates LOEC 300 μM (Evans et al., 2022); IC_{50} binding 7745 uM [6]	Negative [7]	ND	**active**
	**PPAR y**	Yes, activates LOEC 300 μM [5]	Negative [7]	ND	inactive
	**TR β**	ND	ND	ND	/
**PFHxS (355-46-4)**	**AR**	EC_{50} 100 μM (Kjeldsen & Bonefeld-Jørgensen, 2013) IC_{50} 30 μM [8]	No effect [2] inactive AR EcoScreen [3]	ND	**active**
	**ER α**	Inactive (LOEC 200 μM) [5]	No effect [2]	ND	inactive
	**ER β**	Binding Kd 118,71 ± 4,58 μM, likely antagonist in silico [4]	No effect [2]	ND	inactive
	**GR**	ND	ND	ND	/
	**PPAR α**	Yes, LOEC 300 μM [5] Activated at 100 μM [9] Active [10] IC_{50} binding 104 +/- 1,71 μM [6]	Negative [7]	ND	**active**

	**PPAR y**	Yes, LOEC 300 μM [5]<br>Positive significance at 100 μM [7]	ND	ND	**active**
	**TR β**	ND	ND	ND	/
**PFOS (1763-23-1)**	**AR**	Potentiates the stimulatory effect of DHT 50μM + [2]<br>EC_{50} 50 μM [8]<br>IC_{50} 4,7 μM [8]	No effect [2] inactive AR EcoScreen [3]	inactive	**active**
	**ER α**	Activates in the presence of E2, at 50 - 100 μM [2]<br>Active 1 nM + [11]<br>Binding Kd 56.29 ± 3.40 μM, likely antagonist in silico [4]<br>Active [5]	Inactive [12]<br>No effect [2]	**active**	**active**
	**ER β**	Activates in the presence of E2, at 100 μM [2]<br>EC_{50} 29 μM [8]	inactive [12]<br>No effect [2]	**active**	**active**
	**GR**	Increases signal in presence of cortisol [13]	ND	inactive	inactive
	**PPAR α**	Yes, LOEC 300 μM [5]<br>Activated at 250 μM [9]<br>Active [10]<br>Active [14]<br>IC_{50} binding 237 ± 2.55 μM [6]	ND	ND	**active**
	**PPAR y**	Yes, LOEC 100 μM [5]<br>Active [14]<br>EC_{20} 21.19 μM activator in cell based assay [15]	ND	ND	**active**
	**TR β**	ND	ND	ND	/
**PFOSA (754-91-6)**	**AR**	Active [5]	ND	active	**active**

	**ER α**	ND	ND	active	**active**
	**ER β**	ND	ND	active	**active**
	**GR**	ND	ND	inactive	inactive
	**PPAR α**	ND	inactive [5]<br>Nothing significant up to 500 μM [16]	ND	inactive
	**PPAR γ**	ND	inactive [5]<br>Nothing significant up to 500 μM [16]	inactive	inactive
	**TR β**	ND	ND	ND	/
**PFBA (375-22-4)**	**AR**	Potentiates DHT in antagonistic testing [2]	No effect [2]<br>inactive [17]	ND	inactive
	**ER α**	ND	No effect [2]	ND	inactive
	**ER β**	Binding Kd 192.14 ± 4.82 μM, likely antagonist in silico [4]	No effect [2]	ND	inactive
	**GR**	ND	ND	ND	/
	**PPAR α**	IC_{50} binding 3224 μM [6]<br>yes [17]	ND	ND	inactive
	**PPAR γ**	ND	inactive [17]	ND	inactive
	**TR β**	ND	ND	ND	/
**PFPeA (2706-90-3)**	**AR**	ND	no [17]	ND	inactive
	**ER α**	ND	ND	ND	/
	**ER β**	ND	ND	ND	/
	**GR**	ND	ND	ND	/
	**PPAR α**	IC_{50} binding 3279 μM [6]<br>Active, significance at 30 μM [17]	Nothing significant up to 500 μM [16]	ND	**active**
	**PPAR γ**	Active, significance at 100 μM [17]	Nothing significant up to 500 μM [16]	ND	**active**
	**TR β**	ND	ND	ND	/

**PFHxA (307-24-4)**	**AR**	Potentiates DHT [2]	No effect [2]<br>inactive AR EcoScreen [3]<br>inactive [17]	inactive	inactive
	**ER α**	ND	No effect [2]	inactive	inactive
	**ER β**	Binding Kd 147.93 ± 4.45 μM, likely antagonist in silico [4]	No effect [2]<br>Inactive [17]	inactive	inactive
	**GR**	ND	ND	inactive	inactive
	**PPAR α**	Yes, activates [5]<br>LOEC 300 μM<br>Active [10]<br>IC_{50} binding 904 ± 11.54 uM [6]<br>yes [17]	Negative [7]	ND	**active**
	**PPAR y**	Yes, LOEC 300 μM [5]<br>weak activator in cell based assay [15]<br>yes [17]<br>positive [7]<br>positive [7]	ND	inactive	**active**
	**TR β**	ND	ND	ND	/
**PFHpA (375-85-9)**	**AR**	ND	inactive [17]	**active**	**active**
	**ER α**	ND	ND	inactive	inactive
	**ER β**	ND	ND	**active**	**active**
	**GR**	ND	ND	inactive	inactive
	**PPAR α**	IC_{50} binding 275 ± 1.62 μM [6]<br>Active, significance at 1000 μM [17]	ND	ND	**active**
	**PPAR y**	Active, significance at 100 μM [17]	Inactive in cell based assay [15]	inactive	**active**
	**TR β**	ND	ND	ND	/
**PFOA (335-67-1)**	**AR**	EC_{50} 100 μM [8]<br>EC_{50} 11 μM [8]<br>Antagonist at 10 nM [18]	No effect [2]<br>inactive AR EcoScreen [3]<br>No effect [2]<br>inactive AR EcoScreen [3]<br>inactive [17]	inactive	**active**

	**ER α**	Active 100 nM + [11]<br>Binding IC_{50} 469.5 ± 4.6 μM [19] MVLN: signal is increasing 50-200 μM, then decreasing (Both isoforms)<br>Binding Kd 71.95 ± 3.74 μM, likely antagonist in silico [4]<br>Active [5]	No effect [2]<br>Inactive [12]	**active**	**active**
	**ER β**	Activates in the presence of E2, at 100 μM [2]<br>Binding IC_{50} 384.4 ± 3.4 μM [19]	Inactive [12]<br>No effect [2]	**active**	**active**
	**GR**	ND	inactive [17]	inactive	inactive
	**PPAR α**	Yes, activates [5]<br>LOEC 100 μM<br>Activated at 100 μM [9]<br>Active [10]<br>Active [14]<br>Active [20]<br>IC_{50} binding 371 ± 1.62 μM [6]<br>Active, significance at 100 μM [17]	Negative [7]	ND	**active**
	**PPAR y**	Yes, activates [5]<br>LOEC 100 μM<br>Active, significance at 30 μM [17]<br>Binding IC_{50} 370.2 uM; luciferase assay in cells LOEC at 25 μM, agonist [21] confirmed by X-ray [22]<br>EC_{50} 91.12 μM activator in cell based assay [15]<br>Active [14]<br>positive [7]	ND	inactive	**active**
	**TR β**	ND	ND	ND	/

**PFNA (375-95-1)**	**AR**	IC_{50} 52 μM [8]	inactive [8] inactive [17]	inactive	**active**
	**ER α**	Active 100 nM + [11] Active [5]	ND	**active**	**active**
	**ER β**	ND	ND	**active**	**active**
	**GR**	ND	inactive [17]	inactive	inactive
	**PPAR α**	Yes, activates LOEC 300 μM [5] Activated at 100 μM [9] Active [10] IC_{50} binding 277 ± 2.48 μM [6] Active, significance at 100 μM [17]	ND	ND	**active**
	**PPAR y**	Yes, LOEC 300 μM [5] EC_{20} = 24.02 μM activator in cell based assay [15] Active, significance at 100 μM [17]	ND	**active**	**active**
	**TR β**	ND	ND	ND	/
**PFDA (335-76-2)**	**AR**	IC_{50} 6 μM [8]	inactive [8] No effect [17]	inactive	**active**
	**ER α**	Active 100 nM + [11]	ND	**active**	**active**
	**ER β**	ND	ND	inactive	inactive
	**GR**	Increases signal in presence of cortisol [13]	**No effect** [17]	inactive	inactive
	**PPAR α**	Yes, LOEC 300 μM [5] IC_{50} binding 366 ± 6.38 uM [6] Active, significance at 30 μM [17]	ND	ND	**active**
	**PPAR y**	Yes, activates LOEC 300 μM [5] weak activator in cell based assay [15]	ND	inactive	**active**

		Active, significance at 100 μM [17]			
	**TR β**	ND	ND	ND	/
**PFUna (2058-94-8)**	**AR**	ND	inactive [8]<br>No effect in reporter cell line [17]	inactive	Inactive
	**ER α**	ND	Inactive up to 1000 nM [11]	**active**	**active**
	**ER β**	ND	ND	inactive	inactive
	**GR**	ND	No effect in GR CALUX assay [17]	inactive	inactive
	**PPAR α**	$C_{20\ max}$ (human) = 86 μM [23]<br>IC_{50} binding 3265 ± 6.38 μM [6]	No effect on PPAR α activation in reporter cells HepG2 (human LBD)[24]	ND	**active**
	**PPAR y**	ND	inactive (human) cell line [15]	inactive	inactive
	**TR β**	ND	ND	ND	
**6:2 FTOH (647-42-7)**	**AR**	ND	No effect [17]	inactive	inactive
	**ER α**	active [12]<br>Binding Kd 236.14 ± 4.84 μM, likely antagonist in silico [4]<br>Active [5]	ND	inactive	**active**
	**ER β**	active [12]	ND	inactive	**active**
	**GR**	ND	ND	inactive	inactive
	**PPAR α**	ND	inactive [5]<br>Nothing significant up to 500 μM [16]<br>No effect [17]	ND	inactive
	**PPAR y**	ND	inactive [5]<br>Nothing significant up to 500 μM [16]<br>No effect [17]	inactive	inactive
	**TR β**	ND	ND	ND	/

**HFPO-DA (13252-13-6)**	**AR**	Very weak effect [18] Antagonist at 100 μM [18]	inactive AR EcoScreen [3] no effect up to 100 μM, slight antagonism at 300 μM [25]	ND	inactive
	**ER α**	ND	Inactive in binding and MVLN [19]	ND	inactive
	**ER β**	ND	Inactive in binding and MVLN [19]	ND	inactive
	**GR**	BMD for suppression 12549.35 ng/mL [26]	no effect up to 100 μM, slight antagonism at 300 μM [25]	ND	inactive
	**PPAR α**	Yes, activates, LOEC 30 μM [5]	ND	ND	**active**
	**PPAR y**	Yes, LOEC 100 μM [5] Binding IC_{50} N/A; luciferase assay in cells LOEC at 50 μM, agonist [21]	ND	ND	**active**
	**TR β**	ND	ND	ND	/
**8:2 monoPAP (57678-03-2)**	**AR**	ND	No effect on reporter cell line [27] No effect in reporter cell line [17]	ND	inactive
	**ER α**	ND	ND	ND	/
	**ER β**	ND	ND	ND	/
	**GR**	ND	ND	ND	/
	**PPAR α**	ND	No effect in reporter cell line [17]		inactive
	**PPAR y**	ND	No effect in reporter cell line [17]	ND	inactive
	**TR β**	ND	ND	ND	/
**8:2 FTOH (678-39-7)**	**AR**	ND	No effect [17]	Inactive	inactive
	**ER α**	Active LOEC 10 μM [5]	ND	Inactive	**active**
	**ER β**	ND	ND	inactive	inactive
	**GR**	ND	ND	inactive	inactive

	**PPAR α**	ND	No effect [5]<br>No effect [17]	ND	inactive
	**PPAR y**	ND	no effect [5]<br>No effect [17]	inactive	inactive
	**TR β**	ND	ND	inactive	inactive

Legend: ND - no data; / - no decision due to the lack of data available.

References

[1] K. Kolšek, J. Mavri, M. Sollner Dolenc, S. Gobec, S. Turk, Endocrine disruptome - An open source prediction tool for assessing endocrine disruption potential through nuclear receptor binding, J Chem Inf Model 54 (2014) 1254–1267. https://doi.org/10.1021/ci400649p.

[2] A.C. Behr, D. Lichtenstein, A. Braeuning, A. Lampen, T. Buhrke, Perfluoroalkylated substances (PFAS) affect neither estrogen and androgen receptor activity nor steroidogenesis in human cells in vitro, Toxicol Lett 291 (2018) 51–60. https://doi.org/10.1016/J.TOXLET.2018.03.029.

[3] M.P. Carlier, P.H. Cenijn, T. Baygildiev, J. Irwan, S.E. Escher, M.B.M. van Duursen, T. Hamers, Profiling the endocrine-disrupting properties of triazines, triazoles, and short-chain PFAS, Toxicological Sciences (2024). https://doi.org/10.1093/TOXSCI/KFAE131.

[4] Z. Qiu, K. Qu, F. Luan, Y. Liu, Y. Zhu, Y. Yuan, H. Li, H. Zhang, Y. Hai, C. Zhao, Binding specificities of estrogen receptor with perfluorinated compounds: A cross species comparison, Environ Int 134 (2020) 105284. https://doi.org/10.1016/J.ENVINT.2019.105284.

[5] N. Evans, J.M. Conley, M. Cardon, P. Hartig, E. Medlock-Kakaley, L.E. Gray, In vitro activity of a panel of per- and polyfluoroalkyl substances (PFAS), fatty acids, and pharmaceuticals in peroxisome proliferator-activated receptor (PPAR) alpha, PPAR gamma, and estrogen receptor assays, Toxicol Appl Pharmacol 449 (2022) 116136. https://doi.org/10.1016/J.TAAP.2022.116136.

[6] H. Ishibashi, M. Hirano, E.Y. Kim, H. Iwata, In Vitro and In Silico Evaluations of Binding Affinities of Perfluoroalkyl Substances to Baikal Seal and Human Peroxisome Proliferator-Activated Receptor α, Environ Sci Technol 53 (2019) 2181–2188. https://doi.org/10.1021/ACS.EST.8B07273.

[7] A.C. Behr, C. Plinsch, A. Braeuning, T. Buhrke, Activation of human nuclear receptors by perfluoroalkylated substances (PFAS), Toxicology in Vitro 62 (2020) 104700. https://doi.org/10.1016/J.TIV.2019.104700.

[8] L.S. Kjeldsen, E.C. Bonefeld-Jørgensen, Perfluorinated compounds affect the function of sex hormone receptors, Environmental Science and Pollution Research 20 (2013) 8031–8044. https://doi.org/10.1007/S11356-013-1753-3.

[9] F. Sadrabadi, J. Alarcan, H. Sprenger, A. Braeuning, T. Buhrke, Impact of perfluoroalkyl substances (PFAS) and PFAS mixtures on lipid metabolism in differentiated HepaRG cells as a model for human hepatocytes, Arch Toxicol 98 (2024) 507–524. https://doi.org/10.1007/S00204-023-03649-3.

[10] C.J. Wolf, C. V. Rider, C. Lau, B.D. Abbott, Evaluating the additivity of perfluoroalkyl acids in binary combinations on peroxisome proliferator-activated receptor-α activation, Toxicology 316 (2014) 43–54. https://doi.org/10.1016/J.TOX.2013.12.002.

[11] A.D. Benninghoff, W.H. Bisson, D.C. Koch, D.J. Ehresman, S.K. Kolluri, D.E. Williams, Estrogen-Like Activity of Perfluoroalkyl Acids In Vivo and Interaction with Human and Rainbow Trout Estrogen Receptors In Vitro, Toxicological Sciences 120 (2011) 42–58. https://doi.org/10.1093/TOXSCI/KFQ379.

[12] H. Ishibashi, H. Ishida, M. Matsuoka, N. Tominaga, K. Arizono, Estrogenic Effects of Fluorotelomer Alcohols for Human Estrogen Receptor Isoforms α and β in Vitro, Biol Pharm Bull 30 (2007) 1358–1359. https://doi.org/10.1248/BPB.30.1358.

[13] J. Wilson, H.F. Berntsen, K.E. Zimmer, S. Verhaegen, C. Frizzell, E. Ropstad, L. Connolly, Do persistent organic pollutants interact with the stress response? Individual compounds, and their mixtures, interaction with the glucocorticoid receptor, Toxicol Lett 241 (2016) 121–132. https://doi.org/10.1016/J.TOXLET.2015.11.014.

[14] J.P. Vanden Heuvel, J.T. Thompson, S.R.S.R. Frame, P.J. Gillies, Differential Activation of Nuclear Receptors by Perfluorinated Fatty Acid Analogs and Natural Fatty Acids: A Comparison of Human, Mouse, and Rat Peroxisome Proliferator-Activated Receptor-α, -β, and -γ, Liver X Receptor-β, and Retinoid X Receptor-α, Toxicological Sciences 92 (2006) 476–489. https://doi.org/10.1093/TOXSCI/KFL014.

[15] C. Garoche, A. Boulahtouf, M. Grimaldi, B. Chiavarina, L. Toporova, M.J. den Broeder, J. Legler, W. Bourguet, P. Balaguer, Interspecies Differences in Activation of Peroxisome Proliferator-Activated Receptor γ by Pharmaceutical and Environmental Chemicals, Environ Sci Technol 55 (2021) 16489–16501. https://doi.org/10.1021/acs.est.1c04318.

[16] L. Kashobwe, F. Sadrabadi, A. Braeuning, P.E.G. Leonards, T. Buhrke, T. Hamers, In vitro screening of understudied PFAS with a focus on lipid metabolism disruption, Archives of Toxicology 2024 98:10 98 (2024) 3381–3395. https://doi.org/10.1007/S00204-024-03814-2.

[17] A.K. Rosenmai, C. Taxvig, T. Svingen, X. Trier, B.M.A. van Vugt-Lussenburg, M. Pedersen, L. Lesné, B. Jégou, A.M. Vinggaard, Fluorinated alkyl substances and technical mixtures used in food paper-packaging exhibit endocrine-related activity in vitro, Andrology 4 (2016) 662–672. https://doi.org/10.1111/ANDR.12190.

[18] T. Lu, W. Zheng, F. Hu, X. Lin, R. Tao, M. Li, L.H. Guo, Disruption of zebrafish sex differentiation by emerging contaminants hexafluoropropylene oxides at environmental concentrations via antagonizing androgen receptor pathways, Environ Int 190 (2024) 108868. https://doi.org/10.1016/J.ENVINT.2024.108868.

[19] Y. Xin, X.M. Ren, B. Wan, L.H. Guo, Comparative in vitro and in vivo evaluation of the estrogenic effect of hexafluoropropylene oxide homologues, Environ Sci Technol 53 (2019) 8371–8380. https://doi.org/10.1021/ACS.EST.9B01579.

[20] W. Murase, A. Kubota, A. Ikeda-Araki, M. Terasaki, K. Nakagawa, R. Shizu, K. Yoshinari, H. Kojima, Effects of perfluorooctanoic acid (PFOA) on gene expression profiles via nuclear receptors in HepaRG cells: Comparative study with in vitro transactivation assays, Toxicology 494 (2023) 153577. https://doi.org/10.1016/J.TOX.2023.153577.

[21] C.H. Li, X.M. Ren, L.H. Guo, Adipogenic Activity of Oligomeric Hexafluoropropylene Oxide (Perfluorooctanoic Acid Alternative) through Peroxisome Proliferator-Activated Receptor γ Pathway, Environ Sci Technol 53 (2019) 3287–3295. https://doi.org/10.1021/ACS.EST.8B06978.

[22] J.L. Pederick, R.L. Frkic, D.P. McDougal, J.B. Bruning, A structural basis for the activation of peroxisome proliferator-activated receptor gamma (PPARγ) by perfluorooctanoic acid (PFOA), Chemosphere 354 (2024) 141723. https://doi.org/10.1016/J.CHEMOSPHERE.2024.141723.

[23] C.J. Wolf, J.E. Schmid, C. Lau, B.D. Abbott, Activation of mouse and human peroxisome proliferator-activated receptor-alpha (PPARα) by perfluoroalkyl acids (PFAAs): Further investigation of C4–C12 compounds, Reproductive Toxicology 33 (2012) 546–551. https://doi.org/10.1016/J.REPROTOX.2011.09.009.

[24] A.K. Rosenmai, L. Ahrens, T. le Godec, J. Lundqvist, A. Oskarsson, Relationship between peroxisome proliferator-activated receptor alpha activity and cellular concentration of 14 perfluoroalkyl substances in HepG2 cells, Journal of Applied Toxicology 38 (2018) 219–226. https://doi.org/10.1002/JAT.3515.

[25] J.M. Conley, C.S. Lambright, N. Evans, M.J. Strynar, J. McCord, B.S. McIntyre, G.S. Travlos, M.C. Cardon, E. Medlock-Kakaley, P.C. Hartig, V.S. Wilson, L.E. Gray, Adverse Maternal, Fetal, and Postnatal Effects of Hexafluoropropylene Oxide Dimer Acid (GenX) from Oral Gestational Exposure in Sprague-Dawley Rats, Environ Health Perspect 127 (2019) 37008. https://doi.org/10.1289/EHP4372.

[26] F. Zhang, L. Liu, J. Hu, H. Fu, H. Li, J. Chen, C. Yang, Q. Guo, X. Liang, L. Wang, Y. Guo, J. Dai, N. Sheng, J. Wang, Accumulation and glucocorticoid signaling suppression by four emerging perfluoroethercarboxylic acids based on animal exposure and cell testing, Environ Int 178 (2023) 108092. https://doi.org/10.1016/J.ENVINT.2023.108092.

[27] A.K. Rosenmai, F.K. Nielsen, M. Pedersen, N. Hadrup, X. Trier, J.H. Christensen, A.M. Vinggaard, Fluorochemicals used in food packaging inhibit male sex hormone synthesis, Toxicol Appl Pharmacol 266 (2013) 132–142. https://doi.org/10.1016/J.TAAP.2012.10.022.
